# Who Pays for Open Access?

**DOI:** 10.1371/journal.pbio.0020105

**Published:** 2004-04-13

**Authors:** Helen Doyle, Andy Gass, Rebecca Kennison

## Abstract

Publication fees are not borne purely by authors, but are shared by the many organizations whose missions depend on the broadest possible dissemination and communication of scientific discoveries

In the wake of declarations supporting open access to research literature from international bodies including the Organization for Economic Cooperation and Development (OECD) and the United Nations' World Summit on the Information Society (WSIS), advocates and critics of the movement appear to have agreed that the issue warrants a robust, ongoing dialogue—a development undoubtedly in the interest of the scientific community, regardless of its ultimate outcome.

To the extent that listserv messages, editorials, and conference presentations are representative of more widespread reactions to the debate, there appear to be a number of common misconceptions about what open access is and what problems it can or cannot solve. Over the next few months in *PLoS Biology*, we plan to explore the more pervasive of these misunderstandings, in an effort to expose the real challenges that need to be overcome and to identify some possible solutions. Here we address the first of these—the perception that the publication-charge model puts an unfair burden on authors. Subsequently, we will address concerns about the long-term economic viability of the open-access model, the integrity and quality of work published in open-access journals, and the effect that open access will have on scholarly societies.

## Publication Charges—Nothing New

By charging authors a fee to have their work published in lieu of charging readers to access articles, open-access publishers such as the Public Library of Science (PLoS) and BioMed Central (BMC) have transformed the traditional publishing system. This reliance on a seemingly untested revenue stream has generated skepticism that authors will be both willing and able to pay publication charges.

Publication fees are not a phenomenon born of the open-access movement. Many authors regularly pay several thousands of dollars in page charges, color charges, correction costs, reprint costs, and other fees to their publisher, even when such costs are entirely voluntary. In the *EMBO Journal*, for example, authors are allowed six pages of text free, but are then charged $200 per page beyond that. A review of recent issues shows that almost all authors exceed six pages, voluntarily paying on average over $800 to publish their articles.

Furthermore, in addition to paying other publication charges, authors may be willing to pay extra for their articles to be made open access, as several publishers have recently recognized. A recent survey of authors in the *Proceedings of National Academy of Science* (*PNAS*) found that although *PNAS* already makes its content freely available after six months, nearly 50% of *PNAS* authors expressed a willingness to pay an “open-access surcharge” of $500 or more to make their papers available for free online immediately upon publication—this above and beyond the $1,700 in page charges that the average *PNAS* author already pays (Cozzarelli et al. 2004).

Although we recognize that authors who submit to *PLoS Biology* may well be a self-selected group of enthusiastic open-access supporters, we have found that nearly 90% of those who submit manuscripts do not request a fee waiver, and the few who do still offer to pay some portion of the fee.

The concern about authors' ability to pay publication charges will become less pressing as governments, funding organizations, and institutions increasingly support open-access publication on their researchers' behalf. More funding agencies are joining the Howard Hughes Medical Institute, the Wellcome Trust, and others who have already designated funds for open-access publication. (For more information about these funders' announcements and other international policy statements relevant to open access, see http://www.plos.org/openaccess.)

Universities, too, are supporting open access directly by setting aside funds for open-access publication through institutional memberships with BMC and PLoS or through discretionary funds that faculty can tap into to pay publication charges. Such approaches reduce authors' reliance on individual grants to support charges directly and ensure equal access to publishing options that require such payments.

## The Disenfranchised

Even with the steady increase in sources to pay publication fees, detractors claim that open-access publishing may lead to a situation in which some authors are simply unable to publish their work due to lack of funds. The response to this concern is that the ability of authors to pay publication charges must never be a consideration in the decision to publish their papers. To ensure that this happens, PLoS has a firewall in place such that neither the editors nor the reviewers know which authors have indicated whether or not they can pay. Because all work judged worthy of publication by peer review should be published, any open-access business model should be designed to account for fee waivers, just as publishers have always absorbed some authors' inability to pay page and color charges. PLoS grants full or partial publication-charge waivers to any author who requests them, no questions asked.

In part, the savings to institutions, hospitals, nongovernmental organizations, and universities provided by open-access publications could help to establish funds for researchers who are less well supported. In the developing world, as free online access to scientific literature is increasingly seen as a political imperative, organizations such as the World Health Organization, the Oxford-based International Network for the Availability of Scientific Publications, and Brazil's SciELO are likely to become more willing to pay open-access publication charges for authors who cannot afford them. The Open Society Institute (OSI) already pays such costs for universities and other organizations in a number of countries in which the foundation is active by way of a PLoS Institutional Membership that grants waived publication charges to authors while providing compensatory revenue for PLoS.

Perhaps the real misconception about the unfair burden that open access places on authors resides in the terminology—the term “author charge” is itself misleading. Publication fees are not borne purely by authors, but are shared by the many organizations whose missions depend on the broadest possible dissemination and communication of scientific discoveries. Some of those may provide funding for open-access publication as intermediaries between authors and journals, as OSI does. Others—including many government-financed funding agencies—do so directly through their research grants to scientists. In both cases, funding open access is an effective way to fulfill mandates for public access to and accountability over scientific research and to ensure that all worthy research is published.
